# Potential and limitations of epitope mapping and molecular targeting in Hymenoptera venom allergy

**DOI:** 10.3389/falgy.2023.1327391

**Published:** 2023-12-14

**Authors:** Luís Gustavo Romani Fernandes, Edzard Spillner, Thilo Jakob

**Affiliations:** ^1^ Experimental Dermatology and Allergy Research Group, Department of Dermatology and Allergology, University Medical Center Gießen-Marburg, Justus Liebig University Gießen, Gießen, Germany; ^2^ Laboratory of Translational Immunology, Internal Medicine Department, School of Medical Sciences, State University of Campinas, Campinas-SP, Brazil; ^3^ Immunological Biotechnology, Department of Biological and Chemical Engineering, Aarhus University, Aarhus, Denmark

**Keywords:** Hymenoptera venom allergy, epitope mapping, molecular targeting, immunoglobulin E, allergen peptides

## Abstract

Hymenoptera venom (HV) allergy can lead to life threatening conditions by specific IgE (sIgE)-mediated anaphylactic reactions. The knowledge about major allergens from venom of different clinically relevant species increased in the last decades, allowing the development of component-resolved diagnostics in which sIgE to single allergens is analysed. Despite these advances, the precise regions of the allergens that bind to IgE are only known for few HV allergens. The detailed characterization of IgE epitopes may provide valuable information to improve immunodiagnostic tests and to develop new therapeutic strategies using allergen-derived peptides or other targeted approaches. Epitope-resolved analysis is challenging, since the identification of conformational epitopes present in many allergens demands complex technologies for molecular analyses. Furthermore, functional analysis of the epitopeś interaction with their respective ligands is needed to distinguish epitopes that can activate the allergic immune response, from those that are recognized by irrelevant antibodies or T cell receptors from non-effector cells. In this review, we focus on the use of mapping and molecular targeting approaches for characterization of the epitopes of the major venom allergens of clinically relevant Hymenoptera species. The screening of the most relevant allergen peptides by epitope mapping could be helpful for the development of molecules that target major and immunodominant epitopes blocking the allergen induced cellular reactions as novel approach for the treatment of HV allergy.

## 1. Introduction

Insect stings are one of the three most common elicitors of anaphylaxis, along with drugs and food (1), and account for 1.5%–34.1% of cases of anaphylactic shock occurring per year worldwide (2). Among these insects, those of the order Hymenoptera (Aculeata), represented by bees (family Apidae), wasps (family Vespidae) and ants (family Formicidae), stand out (3, 4). In Europe, Hymenoptera stings are among the most frequent causes of severe anaphylaxis in adults (5), and the prevalence of systemic reactions ranges from 0.3% to 7.5% in the European population (6) and from 0.5% to 3.3% in the United States (7).

Clinical history of allergic reactions to stings, skin testing and laboratory tests for the detection of specific IgE (sIgE) against whole venom preparations or individual venom allergens are the basis of routine diagnosis of Hymenoptera venom allergy (HVA) (8). For decades, sIgE tests based on the use of unfractionated venom preparations were the gold standard for diagnosis (9). Despite its widespread use, limited diagnostic sensitivity and cross-reactivity between venoms of different species compromise its accuracy in diagnosing HVA.

Component-resolved diagnostics (CRD), based on the use of individual allergens, previously identified by molecular “omics” approaches (insect venomics), helped to overcome the problems related to the use of venom extracts (10). The official systematic nomenclature of allergens describes 78 venom components of Hymenoptera (www.allergen.org). These components include, among others, various phospholipases A1 and -A2 (PLA2), hyaluronidase, antigen 5, dipeptidyl peptidase IV (DPPIV), phosphatases, icarapin and vitellogenins (11).

Although CRD has provided significant improvements in the diagnosis of HVA, important limitations still exist. Among them, the limited sensitivity of existing allergen panels, diagnostic gaps, and peptide-based cross-reactivity are some of the issues that should be taken into consideration (12). In addition, the absence of commercial availability of important allergens (11), and the possible reactivity to isoallergens and allergen variants to which allergic patients may react (13) negatively affect CRD approaches. Moreover, it is still unclear whether the different CRD profile will be sufficient to predict the success of venom immunotherapy (VIT) or whether it will be able to guide personalized treatment (8, 14).

The identification of the specific regions of the allergen that bind to lymphocyte receptors (BCR and TCR), known as epitopes or antigenic determinants, may help to overcome the limitations of CRD and improve the molecular diagnosis of HVA. Furthermore, the identification of the reactive parts of the allergen may bring new perspectives for the development of novel immunodiagnostic tests using synthetic peptides (15), peptide-based immunotherapy (16), and other molecular targeting approaches such as blocking monoclonal antibodies (17). However, accurate identification of epitopes is not an easy task, as most of the allergens are folded protein structures leading to the existence of several conformational epitopes. To access these epitopes, expensive and complex laboratory techniques such as nuclear magnetic resonance technology, complex mutagenesis studies or hydrogen-deuterium exchange mass spectrometry become necessary for the correct identification of conformational peptides. Unfortunately, none of these techniques are useful for large-scale clinical implementation or for high-throughput, high-content analysis (18, 19).

This review focuses on the use of molecular mapping and targeting approaches for the characterization of the epitopes of major venom allergens of clinically relevant Hymenoptera species, addressing their potentials and limitations for the improvement of diagnosis and therapy for HVA.

## 2. Epitopes mapped in the clinically relevant Hymenoptera venom species

**Figure 1:**
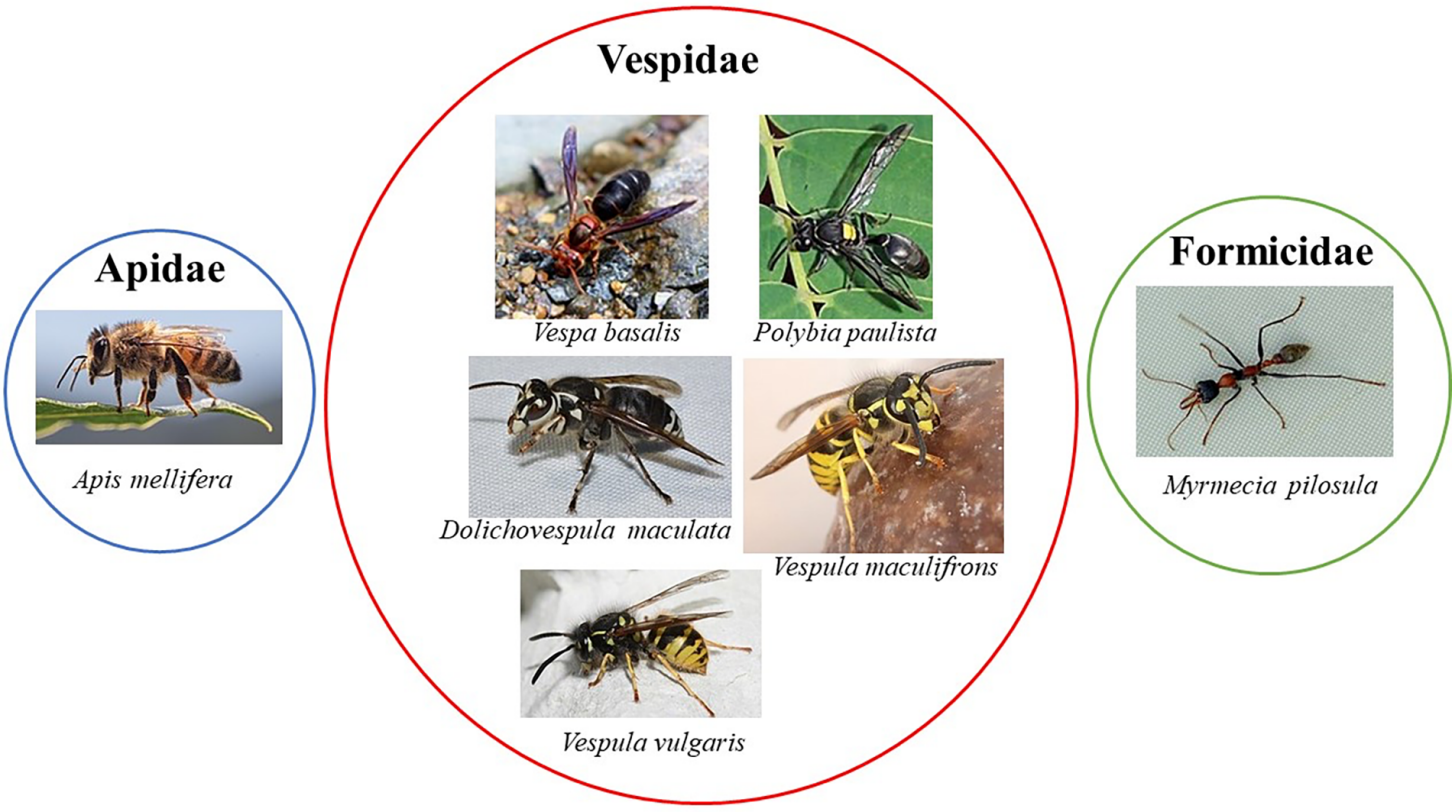
Clinically relevant Hymenoptera species with at least one allergen with epitopes mapped. All the images of the insects were obtained from Wikimedia Commons website (https://commons.wikimedia.org). The images from *Apis mellifera*, *Vespa basalis*, *Polybia paulista*, and *Vespula maculifrons* are under creative commons license—https://creativecommons.org/licenses/by-sa/4.0/deed.en; the images from *Dolichovespula maculate* and *Myrmecia pilosula* are under creative commons license—https://creativecommons.org/licenses/by/2.0/deed.en and the image from *Vespula vulgaris* are under creative commons license https://creativecommons.org/licenses/by-sa/3.0/. The authors from the respective insects images are: *Apis mellifera*—Andrea Fabiani Ph; *Vespa basalis—*nature.Catcher (https://www.Flickr.com/people/96826734@N08/), *Polybia paulista*—Douglas Oliveira, *Dolichovespula maculate*—Andy Reago & Chrissy McClarren, *Vespula maculifrons*—Peterwchen, *Vespula vulgaris*—Magne Flåten, *Myrmecia pilosula—*gailhampshire (https://www.Flickr.com/people/43272765@N04).

The Immune Epitope Database (IEDB) (www.iedb.org) is a free online resource that provides information on experimentally mapped B- and T-cell epitopes studied in humans and other animal species in the context of infectious diseases, allergy, autoimmunity and transplantation. As of June 29, 2023, the IEDB had in its catalog 1,589,806 peptide and 3,182 non-peptide epitopes, of which 11,014 were related to allergic diseases and only 242 were derived from 13 antigens of the Aculeata group (20). Although there is only a small fraction of the epitopes derived from Hymenoptera venom allergens mapped, one must consider that about 30% of the articles do not reach the inclusion criteria of the curation process and are therefore not included in the database (21). To address the information on the already mapped epitopes of the main venom allergens of clinically relevant Hymenoptera species (Figure 1), we consider here only those that have had their reactivity experimentally proven, not considering those only predicted in *in silico* prediction tools. In this sense, we used data obtained from the IEDB and from the literature that showed consistent experimental evidence of epitope reactivity. We present here in Tables 1–3 the linear, discontinuous, and non-peptide epitopes of 16 antigens from species of the Apidae (Apamin, Phospholipase A2 precursor, Hyaluronidase precursor, Melittin, Icarapin, NADH dehydrogenase subunit 2, Phenethyl caffeate, Prenyl caffeate), Vespidae (Phospholypase A1, Hyaluronidase, Mastoparan B, Vespula kinin 1 and Antigen 5 from *D. maculate*, *V. vulgaris* and *P. paulista*) and Formicidae (Pilosulin-1 precursor) groups, respectively.

**Table 1:** Epitopes of the venom allergens from species of Apidae family.

Allergen (IUIS/WHO)	Antigen name	Type and number	Assay type and description	Reactivity
*Apis melifera*				
–	Apamin	linear—1 (22)	radio immuno assay (RIA), qualitative binding	mice
Api m 1	Phospholipase A2 precursor	linear—107 (23–39)	^3^H-thymidine Proliferation (24, 27, 28, 30, 33, 35, 36), purified MHC competitive fluorescence (26, 34, 38, 39) cytokine release: IL-2 (24, 27, 37), IFN-γ (24, 25), IL-4 (24, 25), IL-5 (24, 25), IL-10 (25) and IL-13 (24, 25), biological activity antibody help (25) and neutralization (32), ELISA (29, 32), *in vivo* assay decreased disease (24, 28–30, 40), Immunodot to sIgE (31)	mice (27, 32, 35, 37) and human (23–26, 28–31, 33, 34, 36, 38, 39)
		discontinuous—1 (32, 41)	ELISA and inhibition by antigen—qualitative binding	human
		non peptidic—2. (42–46)	ELISA (43, 44, 46), Western blot (43–45) and inhibition by antigen (42, 43, 46)—qualitative binding	human (43, 44, 46) rabbit (42–45), and rat (43)
Api m 2	Hyaluronidase precursor	discontinuous-1 (47)	cross blocking qualitative binding, ELISA, qualitative binding, x-ray crystallography 3D structure	mice
Api m 4	Melittin	linear– 9 (48–52)	ELISA qualitative binding (48, 50) inhibition by antigen, qualitative binding (49, 50). In vivo assay decreased disease (51) immunoprecipitation qualitative binding (48), ^3^H-Thymidine Proliferation (49, 51, 52)	mice
Api m 10	Icarapin	linear—1 (53)	macroarray sIgE	human
–	NADH dehydrogenase subunit 2	linear—1 (54)	cellular MHC/direct/fluorescence qualitative binding	mice
–	Phenethyl caffeate	non peptidic—1 (55)	*in vivo* skin test—DTH^a^	human
–	Prenyl caffeate	non peptidic—1 (55)	*in vivo* skin test—DTH^a^	human

^a^ DTH, Delayed-Type Hypersensitivity.

**Table 2:** Epitopes of the venom allergens from species of Vespidae family.

Allergen (IUIS/WHO)	Antigen name	Type and number	Assay type and description	Reactivity
*Dolichovespula maculate*				
Dol m 5	Antigen 5	linear—20 (51, 56)	ELISA (51) ^3^H Thymidine proliferation (56)	mice
*Vespula vulgaris*				
Ves v 1	Phospholypase A1	linear—9^a^	multimer/tetramer qualitative binding	human
Ves v 2	Hyaluronidase	linear—46 (57, 58)^a^	western blot qualitative binding (58)	rabbit (58)
			^3^H-thymidine Proliferation (57) multimer/tetramer qualitative binding^a^	human (57)^a^
Ves v 5	Antigen 5	linear—42 (59, 60)^a^	^3^H-thymidine Proliferation (59), ELISPOT IL-4 release (60) multimer/tetramer qualitative binding^a^	human
*Polybia paulista*				
Poly p 5	Antigen 5	linear—9 (61)	SPOT synthesis assay to IgG and IgE, ELISA and skin Prick test	human
*Vespa basalis*				
–	Mastoparan B	linear-1 (62, 63)	ELISA	mice and rabbit
*Vespula maculifrons*				
–	Vespula kinin 1	linear-1 (62)	qualitative ELISA	mice

^a^ IEDB Submission #1000508 (James E.A. et al. 2012).

**Table 3:** Epitopes of the venom allergens from species of Formicidae family.

Allergen (IUIS/WHO)	Antigen name	Type and number	Assay type and description	Reactivity
*Myrmecia pilosula*				
Myr p 1	Pilosulin-1 precursor	linear 2 (64)	inhibition by antigen qualitative binding cross blocking qualitative binding to IgE	human

### 2.1. Apidae family

We can observe that in the Apidae family (Table 1) there is only one species (*Apis mellifera*) with the epitopes described in the IEDB database. In addition, only eight antigens were considered adopting the aforementioned criteria, among which, only four are officially recognized allergens (Api m 1, Api m 2, Api m 4 and Api m 10). Phospholipase A2 (Api m 1) presented the highest number of mapped epitopes, 107 linear, 1 discontinuous and 2 non-peptide. Api m 4 presented seven linear epitopes, and for Api m 2 and Api m 10 one discontinuous and one linear epitope was described, respectively. In addition, the venom peptide apamin and the mitochondrial enzyme NADH dehydrogenase-2 have one linear peptide mapped each. Finally, there are two non-peptide epitopes described for caffeic acid derivatives present in propolis and beeswax.

#### 2.1.1. Api m 1 (phospholipase A2)

Phospholipase A2 (Api m 1) is an active glycoprotein enzyme with a molecular weight around 16 kDa, accounting for 10%–12% of the dry weight of bee venom [reviewed in (65)]. This protein is composed of 134 amino acid residues with a single N-glycosylation at Asn^13^ (66) and presents about 6–8 disulfide bridges that ensure the correct folding of the protein. The catalytic reaction in which this enzyme acts is a calcium-dependent hydrolysis of 3-sn-phosphoglycerides (67). Api m 1 is considered the most important allergenic component of HBV. Between 57% and 97% of HBV-allergic patients are sensitized to Api m 1, comparing different patient cohorts [reviewed in (68)]. In addition, Api m 1 shows a high sequence identity with homologous proteins found in other species of the genus Apis, e.g., Api d 1 from *A.dorsata* shows 91% sequence identity with Api m 1 with a high cross-reactivity between the two allergens (69).

Regarding Api m 1 T-cell epitopes, Carballido et al. (23) identified three continuous linear epitopes corresponding to the PLA^45–62^, PLA^81–92^, PLA^113–124^ amino acid sequences of this allergen. The authors demonstrated that Api m 1-specific CD4^+^ T cell clones from allergic and non-allergic individuals proliferate *in vitro* when stimulated with PLA peptides presented by irradiated autologous B cells expressing HLA-DP and DQ molecules. T cells from allergic individuals showed a greater proliferative response compared with those from non-allergic individuals. Later studies (24, 40) showed that these same peptides were able to detect a refractory proliferative response and a significant impairment in Th1/Th2 cytokine production in T cells from five allergic patients who underwent VIT with therapeutic HBV for two months (24) and in five patients who underwent VIT with a mixture of these peptides (40). These findings suggest that the use of linear T-cell peptides could be a potential tool to monitor the efficacy of VIT and possible candidates for peptide-based VIT for HBV allergy. However, due to the limited number of patients in these studies, more data and evidence are needed to validate the use of Api m 1 epitopes as markers of VIT efficiency and as possible candidates for peptide-based VIT approaches.

Faith et al. (25), addressed the relationship between PLA2 epitope sequence specificity and its ability to stimulate T cells by demonstrating that altered forms of PLA^81–92^ (YFVGKMYFNLID), in which amino acid substitutions at positions 84, 86, 88 and 89 with alanine rendered the PLA-specific T cell clone non-responsive in the proliferation assay. In addition, the substitution of phenylalanine at position 82 generated an altered peptide ligand that inhibited IL-4 production in the PLA-specific T cell clone and in polyclonal T cells from bee venom allergic patients. This phenomenon seems to be related to a lower MHC class II binding affinity for the altered peptide compared to the native one. On the other hand, Texier et al. (26) demonstrated that point mutations in a short peptide comprising the amino acid sequence 81–97 of PLA2, in which the asparagine at position 89 was replaced by leucine, lead to an increased affinity for the following class II human leukocyte antigens (HLAII) alleles, HLAII DRB1*0301 and DRB3*0301. However, in a previous publication using BALB/c mice sensitized with Api m 1, Texier et al. (27) showed that only four of the eight immunodominant epitopes restricted to MHCII I-E^d^ or I-A2 were able to stimulate antigen-specific T cells. Taken together, these findings suggest that the binding regions of the peptides to the TCR or MHCII may differ significantly from one antigenic determinant to another, hampering the fine delineation of Api m 1T cell epitopes.

To overcome the difficulties of MHC binding affinity to short PLA2 peptides, Kämmerer et al. (28) compared two different approaches to delineate PLA2 epitope mapping, using short (15mer) and long (40–60mer) overlapping peptides. The authors showed that peripheral blood mononuclear cells (PBMCs) previously depleted of CD8^+^ T cells, obtained from ten HBV-allergic patients, showed a dominant reactivity only to the long peptides, mainly targeting the C-terminal peptide PLA^290–134^. Furthermore, the authors suggest that the use of long peptides has the advantage of containing more than one epitope that can bind to different HLA alleles, excluding the need for HLA phenotyping of patients. Corroborating these findings, Fellrath et al. (29) performed a phase I clinical trial using the SCIT protocol in 16 patients with long synthetic peptides, LSP1-60, LSP47-99 and LSP90-134, mapping all 134 amino acids of PLA2. The authors showed an induction of T-cell anergy after the SCIT protocol, observed by inhibition of proliferation, and an increase in IFN-γ and IL-10 cytokines in the T cells from patients' PBMCs stimulated with the peptides. Regarding the serological response, there was an increase of sIgG4 but with no change in the sIgE production.

The use of promiscuous peptides, which bind to different MHCII molecules, could also be an interesting alternative to overcome MHC allele restriction. In this context, Tarzi et al. (30), performed an open-label, controlled study using intradermal doses of a mixture of promiscuous HLA-DR-PLA2 T-cell peptides in twelve patients with mild bee venom allergy. The authors demonstrated that after twelve weeks from the start of the immunotherapy protocol, patients showed a reduction in the magnitude of the late-phase skin reaction following challenge with the allergen and a modification in the profile of antigen-specific T cells. After *in vitro* stimulation with PLA2, T cells showed a reduction in proliferative response and IFN-γ production accompanied by an increase in IL-10 production.

Regarding Api m 1 IgE epitopes, Zahirović et al. (31), using phage display methodology, described 46 peptides selected by the specific anti-Api m1 IgG antibody. From these peptides, the authors mapped the three-dimensional (3D) structure of Api m 1 and defined two major regions 17–24 and 119–124 that possibly represent the epitopes of Api m 1. To test this, the authors used four mimotopes [2 linear (SPPNALGRFLPD, LMGPSEL) and 2 cyclic (CWTDLGRKC, CVDKSKPHC)] fused to the pIII protein of bacteriophage M13 and analyzed their reactivity to sIgE from 12 HBV-allergic patients. The peptides showed a strong reaction to sIgE from all twelve patients. In addition, the CVDKSKPHC peptide was not able to activate basophils from three allergic patients tested. However, it was able to induce IFN-γ, but not IL-5 and IL-13 production in T cells of allergic patients stimulated *in vitro* with this peptide. Although the peptides described in this work were able to generate four mimotopes with high specificity for sIgE from allergic patients, it is possible that additional epitopes of sIgE from Api m 1 exist, since pre-selection of the epitopes displayed by the phage with anti-Api m 1 IgG may not be able to bind to them.

Monoclonal antibodies (mabs) derived from antigen-specific B-cell clones from HBV-sensitized individuals (32, 41, 70) or induced by immunization of experimental models (32, 70) allowed the identification of discontinuous antigenic determinants presented on the Api m 1 allergen. Schneider et al. (70) described a conformational epitope identified by two IgG4 mabs produced by hybridomas derived from mononuclear cells of a beekeeper. The authors showed that mabs, obtained from mice sensitized with Api m 1, did not recognize this epitope, indicating an important difference between the antibodies found in sensitized individuals and those artificially induced by immunization. In addition, mabs and serum antibodies from 14 beekeepers and 14 HBV-allergic individuals recognized both forms (glycosylated and non-glycosylated) of PLA2, suggesting that the carbohydrate residue in PLA2 did not represent an important epitope. Duddler et al. (41) demonstrated by site-directed mutagenesis that lysine residues, especially at position 25, abrogate binding to both mabs, indicating that this epitope probably represents the major antigenic region of B cells. Three years later, the same group demonstrated that mouse mabs, derived from B-cell clones generated by PLA2 immunization, recognized linear and carbohydrate-associated epitopes, which differ from the previously described human mab (32). In this regard, the authors suggested that the epitopes recognized by antibodies generated by artificial sensitization in other species could differ from those recognized by human antibodies, leading to a misinterpretation of the relevant allergen epitopes.

The presence of cross-reacting carbohydrates (CCDs) in Api m 1, presented as N-glycan structures of α1,3 and/or α1,6 fucosylation (42), may lead to misinterpretation in the diagnosis of HVA, since almost more than 20% of allergic patients develop anti-CCD antibodies (71). Prenner et al. (42) demonstrated that anti-PLA2 antibodies, induced in rabbits that were sensitized with the glycosylated form of PLA2, recognized plant glycoproteins that have carbohydrate residues α1,3 fucose, such as bromelain from pineapple. Furthermore, removal of α1,3 fucose from this protein significantly reduced anti-PLA2 antibody binding, indicating that sensitization with the glycosylated form of PLA2 induces anti-CCD antibodies. On the other hand, Bencúrová et al. (43) showed that patients allergic to the most common inhalant allergens or insect venom (bee/wasp) allergy had high levels of fucose-reactive IgE detected in ELISA assays. Corroborating these findings, Tretter et al. (46) showed that 34 of 122 sera from individuals classified as allergic to bee venom (RAST score against bee venom ≥ 2) exhibit significant amounts of glycan-reactive IgE, which also recognizes the α1,3-fucose residue of bromelain. In addition, Pöltl et al. (44) and Jin et al. (45) demonstrated that rabbits immunized with these carbohydrates (45) or with horseradish peroxidase (44), a glycoprotein containing this carbohydrate residue, were able to produce antibodies specific for this non-peptide epitope. Despite the lack of clinical relevance of anti-CCD antibodies for the induction of anaphylaxis to Hymenoptera stings (72), it needs to be addressed if the identification of the epitopes of CCD structures may help to detect the presence of false cross-reacting antibodies that hamper the diagnosis of HBV allergy.

#### 2.1.2. Api m 2 (hyaluronidase)

Bee venom hyaluronidase (Api m 2) is a cross-reacting allergen of bee venom with hyaluronidase from other Hymenoptera species, especially yellow jacket wasps (11). Padavattan et al. (47) described an important continuous epitope detected by a mouse IgG1 mab, which competed for Api m 2 binding with sIgE from HBV-allergic patients. By x-ray structural analysis of the crystal structure in complex with a specific Fab region of the antibody, the authors revealed a conformational configuration of this epitope. This seems to be essential for antibody recognition, since neither the specific mab nor human sIgEs recognized a 15-mer linear synthetic peptide comprising the entire epitope. The authors suggest that this Api m 2 epitope may be a potential target for a rational structure-based modification to generate hypoallergenic variants with low IgE binding, but with a preserved ability to induce sIgGs that compete with the sIgE binding site. However, as Api m 2 sensitization rates range from 28 to 60% in different study populations [reviewed in (68)], the efficacy of VIT protocols using only hypoallergenic Api m 2 variants may not be sufficient for HBV desensitization.

#### 2.1.3. Api m 4 (melittin)

Melittin (Api m 4) is a bee venom peptide with 26 amino acids, although it is considered a minor allergen, patients with sIgE to Api m 4 greater than 0.98 kU/L had more severe reactions to stings and increased skin sensitivity during the build-up phase of VIT (73). Fehlner et al. (52) described one major T-cell epitope, located in the region of amino acid residues 11–19, using melittin fragments and their analogs to stimulate antigen-specific T-cell clones *in vitro*. However, King T. et al. (48), using analogs that differ by stepwise truncation of two residues at the N-terminus of residues 2–10, showed that depletion of this region did not alter the ability of melittin to induce T-cell activation. These authors also demonstrated that, despite analogues preserving the B-cell epitope region (residues 21–26), those with more than two residues deleted at the N-terminus did not induce a significant antibody response in immunized mice. Interestingly, the use of synthetic analogs with the transposed amino acid sequence induced the production of specific antibodies triggered to the transposed peptide but unable to bind to melittin. In addition, T cells from mice immunized with melittin proliferated under stimulation with the transposed analogues (49). In a later work, the same author demonstrated that the major T-cell epitope of Api m 4 (KVLTTGLPALISW) partially suppressed the proliferative T-cell response to melittin in allergen-sensitized mice that were pretreated with this peptide subcutaneously or intranasally (51). Taken together, these results suggest that the T-cell epitopes of Api m 4 play an important role in the induction of the specific immune response to this allergen, especially in the stimulation of antigen-specific T and B cells.

#### 2.1.4. Api m 10 (icarapin)

Icarapin (Api m 10) is an important marker allergen presented in low abundance in HBV (74). Patients with dominant sensitization to this allergen have been associated with a relevant risk factor for HBV VIT failure. An under-representation of this allergen in several commercial HBV therapeutic preparations could be a possible explanation for this fact (75). Macroarray assays using 64 (15-mer) synthetic peptides with 12 overlapping amino acids and serum from HBV-allergic patients allowed the characterization of linear IgE epitopes of this allergen (53). Data from these assays identified 29 Api m 10 peptides recognized by sIgE from individual sera, with one peptide, Api m 10_(160–174),_ showing a positive reaction for all Api m 10-positive sera tested, indicating that this peptide probably is the dominant linear IgE epitope of the Api m 10 allergen. Due to the dominant IgE reactivity that this peptide shows in some individuals (75), there are potential applications in peptide-based immunodiagnostic tests and to be a possible source for increasing the representation of Api m 10 in VIT preparations.

### 2.2. Vespidae family

Regarding the Vespidae family (Table 2), we can observe that only five species of this group (*Dolichovespula maculata, Vespula vulgaris*, *Polybia paulista*, *Vespa basalis* and *Vespula maculifrons*) have some antigen with epitopes already mapped with some experimental evidence. Among them, *Vespula vulgaris* presented the highest number of mapped epitopes, among which: nine linear for phospholipase A1 (Ves v 1), 46 linear for hyaluronidase (Ves v 2) and 42 linear for antigen 5 (Ves v 5). *Dolichovespula maculata* presented 20 linear epitopes mapped to antigen 5 (Dol m 5), while *Polybia paulista* presented nine linear epitopes to this same allergen (Poly p 5). *Vespa basalis* and *Vespula maculifrons* showed one linear epitope for mastoparan peptide and one for vespula kinin 1, respectively.

#### 2.2.1. Ves v 5 (antigen 5)

Ag 5 from *Vespula vulgaris* (Ves v 5) is a 23 kDa protein and consists of 204 amino acids with a high degree of cross-reactivity with Ag 5 from *D. arenaria* and *D. maculata* (76). The T-cell epitopes of this protein were mapped by assessing the T-cell response of allergic individuals in two distinct studies that used different methodological approaches (59, 60). Bohle et al. (59) described 17 linear peptides capable of inducing proliferation of antigen-specific T-cell from patients allergic to wasp venom, as measured by ^3^[H]-thymidine incorporation. In addition, the authors identified a dominant T cell epitope, Ves v 5_(181–192)_, which was not predominant in T cell activation from non-allergic individuals. Interestingly, these activated T cell clones did not produce large amounts of IL-4 (measured in the cell culture supernatants) suggesting that the T cell response to Ves v 5 did not exhibit a Th2 predominance.

On the other hand, Aslam et al. (60) described two major epitopes restricted to HLA-DRB1*1501 that stimulated antigen-specific CD4^+^ T cells *in vitro* from wasp allergic patients. In this work, the authors previously expanded CD4^+^ T cells in cell cultures using stimulation with Ves v 5 recombinant. Afterwards, they examined the cell cultures for cytokine production in ELISpot assays for IL-4 detection. For this, they induced activation of antigen-specific CD4^+^ T cells by stimulation with L cells (mouse fibroblast cell line) transfected with DRB1*1501 and pulsed with the peptide derived from Ves v 5. Furthermore, the use of HLA tetramers loaded with peptides matching the major epitope sequences enabled the detection of epitope-specific T cells from patients undergoing immunotherapy treatment. The authors showed an increase in cells that recognized the peptides presented on HLA tetramers in the induction phase of immunotherapy (first 3–5 weeks), followed by a significant decrease after the seventh week. These results suggest that Ves v 5 major T-cell epitopes may be a useful tool to track changes in antigen-specific T-cell frequency during immunotherapy.

Also related to tetramer-based methods, James E.A. et al. (IEDB database number 1000508) describes four HLA-DR restricted epitopes of Ves v 5 recognized by human CD4^+^ T cells using tetramer-guided epitope mapping (TGEM) approaches, a technique that uses human leukocyte antigen (HLA) class II tetramers for epitope identification through peptide screening procedures (77). However, no functional analysis is available to assess the role of these epitopes in the clinical or functional context.

#### 2.2.2. Ves v 2 (hyaluronidase)

*Vespula vulgaris* hyaluronidase (Ves v 2) is an enzymatically active glycoprotein that presents two isoforms (Ves v 2.01 and Ves v 2.02) that share 58% amino acid identity, with 327 overlapping residues [reviewed in Seppälä et al. (57)]. The cross-reactive carbohydrates (CCDs) presented in this protein and in Api m 2 are responsible for nonspecific binding to IgE, being recognized as the most relevant Hymenoptera venom allergens that exhibit cross-reactivity (58). In this context, Seppälä et al. (57), using 12-mer overlapping peptides spanning the entire amino acid sequence of Ves v 2.01, described 28T-cell epitopes that stimulated *in vitro* proliferation of T-cell lines (TCL) derived from wasp-allergic individuals. Among them, three were found to be the most relevant Ves v 2.01_(4–21)_, Ves v 2.01_(109–126)_, Ves v 2.01_(157–171)_, none belongs to CCDs regions. Furthermore, the authors reported no differences in epitope recognition in TCL generated with the glycosylated and non-glycosylated allergens. On the other hand, Seismann H. et al. (58) demonstrated that the peptides Ves v 2a _(222–235)_, isoform 1, and Ves v 2b _(224–237)_, isoform 2, induced the production of monospecific antibodies in immunized rabbits. These antibodies recognized both natural and recombinant forms of Ves v 2a and Ves v 2b, showing that these peptides induced allergen-specific antibodies unrelated to CCD recognition. Taken together, these results indicate that both isoforms of Ves v 2 have peptidic epitopes that induce antigen-specific T and B cell clones, and that the CCD regions probably are not a relevant antigenic determinant.

Furthermore, the IEDB database records 9 HLA-DR restricted epitopes derived from Ves v 2 and 9 for phospholipase A1 allergens (Ves v 1). James E.A. et al. (IEDB database number 1000508), using TGEM approaches, described the specific binding of human CD4^+^ T cells to these peptides. However, as previously mentioned, the absence of functional or clinical context in this case compromises the correct interpretation of these results.

#### 2.2.3. Dol m 5 (antigen 5)

The white face hornet (*Dolichovespula maculata*) presents antigen 5 (Dol m 5) as the major allergen of its venom (51, 56). King T. et al. (56) mapped the T-cell epitopes of this allergen and found that 20 synthetic peptides with 10-residue overlaps spanning the length of Dol m 5 displayed the ability to stimulate mouse splenic T-cell proliferation in ^3^[H]-thymidine assays. Using six different MHC haplotype mouse strains (C57BL/6, BALB/c, C3H/He, P/J, RII and ASW/sn), previously sensitized with the allergen, they showed that these peptides stimulated antigen-specific T cells from at least one of the mouse strains described above. However, only three peptides were able to stimulate T cells proliferation from five of the six mouse strains, considered therefore as the major T cell epitopes. In addition, one major peptide Dol m 5_(176–195)_ showed cross-reactivity with homologous antigen 5 from *Vespula vulgaris* and *Polistes annularis*. The same authors also demonstrated, one year later (51) that, BALB/c mice treated with three subcutaneous or intranasal doses of the peptides, prior to sensitization with Dol m 5, showed a partial reduction in T-cell proliferation and in the early phase of the antibody response compared to their respective controls. Together, these findings indicate that the three main peptides described above modulate T cell responses in the context of different MHC haplotypes. However, to establish the clinical relevance of these peptides it is necessary to access the ability of Dol m 5-derived peptides to bind to sIgE and to activate T cell clones from patients allergic to *D. maculata* venom.

#### 2.2.4. Poly p 5 (antigen 5)

*Polybia paulista* is a neotropical social wasp present in South America, especially in southeastern Brazil, northern Argentina and Paraguay, and is related to severe accidents and cases of allergic reactions, including anaphylaxis (78). Santos-Pinto et al. (61), using 66 overlapping synthetic peptides corresponding to the complete sequence of *P. paulista* antigen-5 (Poly p 5), described nine linear epitopes that showed positive reactivity to sIgG from a pool of serum from five allergic patients identified by SPOT and ELISA assays. However, only one of them, with the linear amino acid sequence VGHYTQVVWAKTKE, showed positive reactivity to sIgE, confirmed also by the IgE-mediated reaction observed in the Prick test. Although these results indicate the importance of this epitope in the Poly p 5 allergen, other allergens such as phospholipase A1 (Poly p 1) (79) and hyaluronidase (Poly p 2) (80) also play an important role in sensitization to *P. paulista* venom. Further studies, mapping the major epitopes of these allergens, may help in the development of more accurate epitope-based diagnostic tests containing the major immunogenic epitope sequences derived from these three allergens.

#### 2.2.5. Wasp venom peptides

Wasp venom peptides showed important bioactive properties related to antimicrobial, anti-inflammatory, anticoagulant, cytotoxicity to cancer cells, among other physiological effects, with potential application in different areas (81). Mastoparan and mastoparan-like peptides are potent inducers of mast cell degranulation and contribute to the symptoms of pain, inflammation and edema in wasp envenomation (82). Vespula kinin 1 (VSK1) is a 17 amino acid peptide consisting of a bradykinin molecule and eight glycosylated amino acids (83), and is involved in inflammatory and algesic effects (84). In addition, venom peptides such as mastoparan (62, 63) and VSK 1 (62) can induce activation of the immune response in experimental animal models.

Ho et al., using mouse (62, 63) and rabbit (63) sera from mastoparan-sensitized animals in ELISA assays, showed that Mastoparan B from *Vespa basalis*, a 14 amino acid peptide (LKLKSIVSWAKKVL), was recognized by mouse sIgG1 and rabbit sIgG. The authors also demonstrated that substitution of lysine at position four and tryptophan at position nine decreased the antibody binding activity to this peptide. In addition, removal of lysine residues considerably reduced the immunogenic and antibody-binding properties of the peptide (63). The same research group demonstrated that synthetic peptides derived from the VSK1 sequence, isolated from *Vespula maculifrons* venom, induced sIgG1 response in sensitized mice. Carbohydrate-bound peptides, which had galactose residues in tryptophan at position three and four, showed a lower ability to induce the antibody response, suggesting that the carbohydrate portions in VSK1 could interfere, in this case, with the T-cell-dependent humoral immune response (62).

Therefore, epitopes of wasp venom peptides, such as mastoparan and VSK1, may be potential molecular targets for the development of specific blocking ligands, such as mabs, which could help in the future development of new therapeutic approaches for wasp venom envenomation.

### 2.3. Formicidae family

Concerning the family Formicidae (Table 3), the IEDB database described two linear epitopes for the venom precursor peptide pilosulin 1 (Myr p 1) from *Myrmecia pilosula* (Australian jumping ant). Donavan et al. (64), using six synthetic peptides overlapping the C-terminal region of rMyr p 1 in radioimmuno-competition (IgE-RIA inhibition) assays, showed that two of them, corresponding to residues 67–112 and 93–112, exhibited an inhibitory effect on the binding of sIgE from three ant-allergic patients to the allergen rMyr p 1. The peptide of the tested series that showed the highest inhibition was the 93–106 region, a 14 amino acid sequence (KEAIPMAVEMAKSQ). Moreover, as peptides that included less than 14 residues, corresponding to this same region, failed to inhibit antibody binding to the native allergen, conformational factors may likely be involved in their specific binding to sIgE. Despite the evidence of the importance of this region for sIgE recognition, functional properties, such as the ability to induce T cell-mediated immune response and basophil or mast cell activation, need to be addressed to determine the role of this epitope in Myr p 1 sensitization.

Fire ants (genus Solenopsis), especially the species *Solenopsis invicta*, have been reported to induce severe anaphylaxis in sensitized patients, especially in South and Central America, as well as in the Southeastern USA (85). Padavattan et al. (86), evaluating the crystal structure of the major recombinant fire ant allergen, Sol i 3, combined with the immunological properties of monoclonal antibodies directed to Sol i 3 related in a previous study (87), predicted four non-conserved epitopes that are unique to this allergen, with no cross-reactivity with antigen 5 from other Hymenoptera species. However, since there was no further experimental evidence to confirm that these predicted epitopes are the major epitopes of Sol i 3, these results should be carefully interpreted.

## 3. Molecular targeting of epitopes for therapy

As emphasized before, knowledge on epitopes of the allergens will facilitate understanding of their clinical and immunological relevance for diagnostic purposes and as a consequence fuel the establishment of concepts for selective targeting of individual areas or sites on the allergen surface.

Targeting of defined epitopes on venom allergens can prototypically be performed by using antibodies derived from individuals or animals exhibiting immunoreactivity to the allergen. This approach has been applied for several venom allergens and several examples are mentioned above. Alternatively, antibodies nowadays can be obtained by combinatorial approaches, which enable bypassing the immunization and affinity maturation within an animal (58, 88).

One of the early examples is the development of both human and murine monoclonal antibodies against Api m 1, which has been mentioned above (70). However, having such highly defined tools at hands has scarcely resulted in precise atomic description of the corresponding epitopes. An exception is the crystal structure of Api m 2 in complex with a murine antibody fragment (47). Likewise, only limited information on frequency of recognition of certain epitopes by patient IgE and the subsequent functional impact of recognition is available.

Another markable exception is also the description of the CCD pan-epitopes found on the majority of venom allergens. A crystal structure of an antibody fragment in complex with a disaccharide surrogate comprising the innermost GlcNAc and the 1–3-linked fucose revealed insights into the interactions on an atomic level (89). The study also uses CCD-specific monoclonal IgE to prove the biological activity in both activation of effector cells and the facilitation of allergen binding via CD23. While in the case of CCDs the clinical relevance of the epitope is considered low, the diagnostic relevance should not be underestimated. Recombinant allergens are typically devoid of CCD structures, but the use of venom from classical and potentially new insects is often compromised. Hence, having specific reagents targeting defined CCD epitopes helps to prove or disprove the presence of CCD reactivity, in particular for novel venoms and novel food such as grasshoppers, mealworms and crickets. Future food, e.g., Diptera such as the black soldier fly, remain to be assessed critically for CCDs.

The understanding that such venom epitope specific antibodies have a therapeutic potential, has been shaped by early studies showing that passive immunization of HBV-allergic patients with purified IgG from tolerant beekeepers increased the tolerated venom dose and protected against systemic reactions when undergoing rush immunotherapy with HBV (90, 91). Since then, however, antibody technologies have advanced significantly by the strategies for humanization of antibodies and the advent of combinatorial and single cell techniques.

One of the important questions in relation to inhibitory monoclonal antibodies is if a targeting of specific epitopes can provide information on recognition and also clinical relevance, severity of symptoms, and other parameters. Targeting for inhibition of IgE binding by molecules of defined characteristics can be done in a variety of settings, ranging from inhibition ELISA and ImmunoCAP to cellular assays such as mediator release assays from passively sensitized cell lines and basophil activation tests using patient samples. Beyond the *in vitro* assays mentioned, animal models provide *in vivo* evidence of inhibition of allergenic activity. All of these assays can address either single epitopes by single molecules or several epitopes by combinations of molecules or single molecules of combined specificity. However, as most of the described approaches represent sensitization tests only, convincing evidence for clinical relevance remains difficult to find.

The outcome of such approaches, however, could be the information of contribution of epitopes to the collective allergenic potential. In the case of the peculiar dominant epitope of Api m 10, a significant portion of IgE reactivity towards the epitope has been shown (53). Nevertheless, the contribution to the allergenic activity remains to be verified.

Factors that might affect the relevance of epitope-specific recognition are intrinsic to the individual antibodies such as affinity and architecture of the resulting allergen/antibody complex. In addition, the complexity of the entire IgE repertoire and the presence of other isotypes with identical specificity in individual patients might be of utmost diagnostic interest. Moreover, the plasticity of the IgE and the IgG repertoire prior to and upon treatment would be highly interesting to know, since the epitopes in tolerant individuals might reveal option for monitoring and therapeutic intervention. Hence, targeting of venom allergens in an epitope-specific manner is demanding in several aspects.

Recently, the therapeutic use of monoclonal antibodies for blocking of allergens has been advanced by combinations of 2 or 3 antibodies specific for Fel d 1 and Bet v 1, providing efficient protection against effector cell activation and symptoms in patients for several weeks (17, 92, 93). These IgG4 antibodies were specifically selected for efficient blocking IgE binding to their respective epitopes. It is of importance that the 15 kDa allergens Bet v 1 and Fel d 1 demand 2–3 antibodies for sufficient blocking. It remains open if larger allergens require larger numbers of blocking molecules. Recently, a set of 4 blocking antibodies against Ara h 2 has shown efficacy in animal models, with Ara h 2 being 3–4 times larger than Bet v 1 (94). Food allergens, however, are likely to have different types and numbers of dominant epitopes as compared to venom allergens.

Mechanistically, the antibodies are designed to block the interaction of IgE on effector cells with their specific epitopes, hence the recognition of identical or proximal epitopes and the affinity are crucial for efficacy. Recently, the impact of affinity for blocking efficacy has been shown for antibody derivatives of high and low affinity to the apple allergen Mal d 1 (95). Beyond blocking IgE recognition, effector functions such as engagement of inhibitory Fc receptors such as FcγRIIb, prevention of CD23-mediated facilitated allergen binding, and others have been discussed. The role of the IgG subclass in blocking efficacy is still not fully understood (96).

Such antibodies with blocking potential, in allergy as well as infectious diseases, are typically obtained from humans with documented IgG reactivity against the target structure, but also from wild type and humanized mice and other advanced technical approaches. Pure *in vitro* antibody technologies relying on synthetic antibody libraries have been used in the context of HVA. While the approach of generation of antibodies is irrelevant, the capability of targeting important epitopes is crucial.

**Figure 2:**
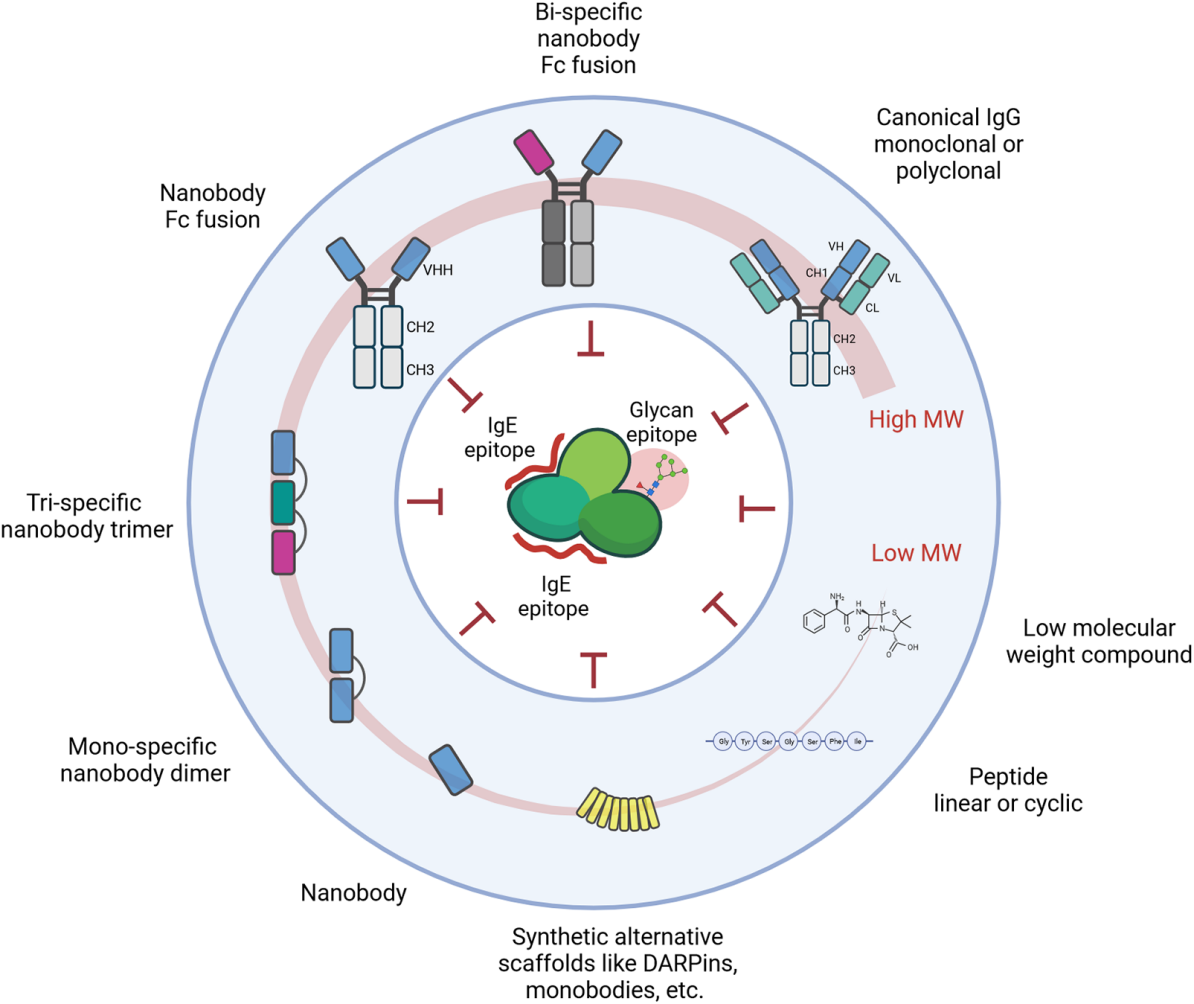
Schematic representation of molecules and approaches targeting and blocking defined epitopes on the surface of allergens. These epitopes primarily include IgE epitopes, but also IgG epitopes or pan-epitopes such as CCD moieties.

The underlying principle of all approaches remains the targeting of important IgE epitopes which hamper recognition by the endogenous effector cell machinery (Figure 2).

Any of the molecules developed over the last decades could represent a potentially efficient approach. This extensive list includes but is not limited to antibody formats beyond the prototypical IgG1, alternative scaffolds like monobodies, affibodies, darpins, lipocalins, and even non-proteinic molecules like aptamers.

Nevertheless, factors that render some of the formats more likely are the size of the interface area and the functional affinity. The larger the area blocked and the higher the affinity, the more likely it is that the targeting moiety competes with or blocks IgE binding.

Recently, smaller derivatives of antibodies with footprints of similar size have entered the stage in allergology. Nanobodies or single domain antibodies are the antigen-binding region in heavy chain only antibodies from camelid species (97). They comprise a single domain of 15 kDa with 3 CDRs only, involving predominantly the CDR3 for antigen interactions (98, 99), which is on average longer than the CDR3 of conventional antibodies (98, 100). The long CDR3 loop, the prolate shape of nanobodies, and the involvement of framework residues result in a convex paratope that can reach into clefts (e.g., active sites and binding pockets) and bind with high affinity epitopes less accessible for conventional antibodies (99) and with similar size as those recognized by canonical antibodies with 2 binding domains. The small size together with the stability and a general absence of immunogenicity make nanobodies highly interesting for a plethora of applications in diagnostics and therapeutic intervention.

In the context of allergic diseases, the use of blocking nanobodies for therapeutic purposes has been recently highlighted (101) and the generation of allergen-specific nanobodies has subsequently provided the initial corner stone for assessment of potential in allergy-related applications. Nanobodies have been generated against not only pollen allergens (97, 102), and food allergens (103), but also venom allergens (104) and downstream formats like multimeric nanobodies and nanobody fusion proteins in the format of IgE have highlighted the ease of nanobody technologies.

Initial data on nanobodies with specificity for allergens such as Bet v 1 have recently provided clear proof-of-concept for the selective inhibition of IgE binding to the corresponding epitopes (102). Notably, the frequency of recognition appears relatively conserved between different patients, pointing to underlying biochemical or immunological cues driving the recognition by IgE and also corroborating the potential for a differential assessment of individual epitopes.

No matter what targeting molecule is used, therapeutic targeting of important epitopes appears to be possible in HVA. Currently open is the need for targeting several epitopes on a larger number of venom allergens as compared to targeting the main epitopes on the abundant allergens such as Api m 1 and Ves v 5. This question, however, is not limited to HVA. Hence, future studies are needed to address the feasibility of molecular targeting in HVA.

## 4. Discussion

The precise identification of allergen epitopes forms the basis for the development of antibody and peptide-based immunotherapies and new immunodiagnostic tests. In this review, we presented the main epitopes, described with some empirical evidence of their reactivity, of the venom allergens from clinically relevant Hymenoptera species.

Firstly, it can be noted that there is a lack of epitope identification of several important allergens from different Hymenoptera species. Regarding HBV, there are only four allergens with their epitopes mapped (Table 1). Important marker allergens such as Api m 3, which allows discrimination between bee and YJ/Polistes venom sensitization, and the major allergen Api m 5 (8) did not show any described epitopes. Moreover, in the family Apidae, only honeybees have any allergen with mapped epitopes. Regarding the families Vespidae and Formicidae, there is also an underrepresentation of the epitopes of the main allergens of clinically relevant species, such as *Vespa crabro*, *Polistes dominula*, *Vespa velutina*, *Solenopsis sp*, among others. Moreover, only *Vespula vulgaris* presented more than one allergen (Ves v 1, Ves v 2 and Ves v 5) with the epitopes mapped (Table 2). One possible reason to this could be the high homology and cross-reactivity in the Hymenoptera venom allergens, as observed between HBV allergens with other species of the genus such as *Apis dorsata* (105), and in a specific allergen as Antigen 5 presented in the venom of Vespoidea Superfamily (106).

However, species from different genera of the Apidae family, such as bumblebees (Bombus), presented a limited cross-reactivity with HBV (only 53% of similarity in the case of PLA2) (107), which could explain the related cases of VIT failure with the use of HBV preparations to treat bumblebee venom sensitized patients (108). On the other hand, highly homologue allergens such as antigen 5, shown sIgE cross-reactivity, for example to YJV and *Polistes dominula* venom (PDV), representing a diagnostic challenge in the areas where both species are endemic (106). Thus, the mapping of the major epitopes of a specific venom allergen from different Hymenoptera species can contribute to define targets of the immunodominant regions in allergens with high sequence identity and in those showing low identity. This approach could be useful to the development of peptide-based diagnostic tests, allowing the correct identification of the culprit species.

Most of the epitopes described for HV allergens were mapped in the 1990s and early 2000s, allowing the identification of mainly linear epitopes capable of stimulating antigen-specific T cells *in vitro* to proliferate or produce effector cytokines and be recognized by allergen-specific immunoglobulins (Tables 1–3). Thus, it is necessary to consider the technical limitations related to the methods that were used to map these epitopes. As reviewed by Potocnakova et al. (19), epitope mapping methods are expensive, labor-intensive, time-consuming and often do not identify all epitopes. For example, the low amount of allergen-specific T cells in the periphery makes it difficult to detect the specific epitope response (109). To overcome this, some mapping studies have used pre-expansion of allergen-specific T cells with the whole allergen and IL-2-derived T cell lines (25) from allergic patients (57). The problem with these approaches, apart from the laborious and time-consuming procedures, is that they may be unable to expand all T-cell clones that have evolved to recognize a few minor epitopes, allowing only the expansion and selection of high-response clones.

In this context, there are probably an underestimation of the real number of the epitopes of a specific allergen, especially in the identification of conformational epitopes. It is believed that the most of identified linear sequences of the B-cell antigenic determinants is indeed parts of conformational epitopes, since more than 70% of the discontinuous epitopes are composed of 1–5 linear segments with 1–6 amino acids of length. In addition, conformational epitopes play an essential role in the recognition of Hymenoptera venom allergens, since allergen-specific antibodies recognize most of venom proteins in their native state, while binding is reduced when these proteins are unfolded. Furthermore, antibodies that detect unfolded proteins are more associated with cross-reactions between allergens from different Hymenoptera species than those that recognize native proteins [reviewed in (110)].

However, the precise identification of conformational epitopes requires more complex and sophisticated techniques like, mass spectrometry X-ray crystallography, nuclear magnetic resonance or phage display libraries [reviewed in (19, 111)]. Even using techniques, like phage display libraries there are limitations in the identification of the conformational epitopes (31, 111). As mentioned before, the pre-selection of the phage-displayed epitopes with anti-allergen specific IgG antibodies could not be able to identify all the sIgE binding epitopes (31). Moreover, these approaches cannot detect alterations in allergens caused by post-translational modifications. Additionally, the determination of the actual position of the epitope on the allergen structure can be limited, since the identification of the conformational epitopes is generally based only in the assignment of the mimotopes sequences with the allergen 3D structure using predictive web tools [reviewed in (111)].

Determining the actual impact that the major epitopes of a specific allergen have on the patient's allergic response to HV is one of the key challenges regarding epitope-mapping approaches. Unfortunately, there are few data in the literature relating the use of the epitopes described for HV to any clinical practice for HVA. About HBV, there are three phase I clinical trials using peptides derived from Api m 1 T-cell epitopes (29, 30, 40). As discussed earlier in this review, these clinical trials have shown the ability of these peptides to modulate the T-cell response to a non-responsive/tolerogenic profile in treated patients. However, in the study conducted by Müller et al. (40), it seems that only a partial protection can be induced with these approaches, since some patients showed strong reactions after the sting challenge, probably due to sensitization to other HBV allergens. As reviewed in Zahirović et al. (112), even though these clinical trials have demonstrated some effect on the modulation of immune mechanisms after the VIT protocol, there is not enough data to guarantee the efficiency of this treatment, and no new trials have been performed after the ones mentioned before.

Taking the example of the first generation of T-cell peptide-based immunotherapy for cat allergy, that used subcutaneous doses of long peptides (27 amino acids) derived from the Fel d 1 allergen, it was shown that, despite the clinical benefits shown by patients undergoing therapy compared to placebo controls, adverse events such as nasal congestion, flushing, pruritus and delayed asthmatic response were observed. In addition, the protocol used showed poor long-term clinical efficacy. However, later studies using mixtures of short peptides derived from Fel d 1 (seven peptides ranging from 13 to 17 amino acids) administrated intradermally, with careful dose adjustments, showed a significant reduction in adverse effects related to the first-generation immunotherapy, as well as prolonged clinical efficacy (two years after the start of immunotherapy) [reviewed in (113)]. In this sense, a similar strategy using mixture of short T-cell peptides derived from the Hymenoptera venom's major allergens should be advantageous for reducing strong reactions and improving the clinical efficacy of new T-cell peptide-based VIT protocols.

In this context, vaccines based on defined B-cell epitopes, which consist of intrinsically non-IgE-reactive peptides derived from the allergen's IgE-binding sites, linked to protein transporters that provide T helper cell activation, may also be a promising strategy for triggering allergen-specific tolerance. The mechanisms involved are related to the induction of sIgG antibodies which inhibit the cross-linking of allergens with sIgE presented on mast cells and basophils, thus preventing immediate allergic inflammation, and also to the suppression of T-cell mediated allergic inflammation by inhibiting IgE-facilitated presentation of the allergen (114).

Assays that allow the assessment of the functional role of the HV allergens' epitopes are crucial to understand their relevance in triggering the immune response. Regarding the B cell epitopes, the measurement of the capacity that the epitopes, from a specific allergen, have to cross-link IgE in the FcεRI receptors of mastocytes or basophils could help to identify and discriminate relevant from irrelevant epitopes. In this sense, the development of basophil or mastocyte activation tests associated with high affinity monoclonal antibodies derived from allergic patients could be an interesting and useful approach (111).

Considering the increasing knowledge about the venom allergens, the structural and immunological basis of recognition, and the nature of IgE and IgG epitopes, the development of epitope-specific diagnostic assays is becoming a realistic scenario. Although the benefit in clinical routine needs to be shown, the benefit for the development of novel therapeutic approaches is evident.
